# Exposure to E-Cigarette Marketing and Susceptibility to Future Vaping among Black and Latino Adolescents in the United States

**DOI:** 10.3390/children11040465

**Published:** 2024-04-13

**Authors:** Ruthmarie Hernández-Torres, Hongyue Wang, Rafael Orfin, Eida M. Castro-Figueroa, Jeffrey Freeman, Ana Paula Cupertino, Deborah J. Ossip, Karen M. Wilson, Francisco Cartujano-Barrera

**Affiliations:** 1 Department of Psychiatry and Behavioral Sciences, Memorial Sloan Kettering Cancer Center, New York, NY 10022, USA; 2 Department of Biostatistics and Computational Biology, University of Rochester Medical Center, Rochester, NY 14642, USA; hongyue_wang@urmc.rochester.edu; 3 Department of Public Health Sciences, University of Rochester Medical Center, Rochester, NY 14642, USA; rafael_orfin@urmc.rochester.edu (R.O.); deborah_ossip@urmc.rochester.edu (D.J.O.); francisco_cartujano@urmc.rochester.edu (F.C.-B.); 4 School of Behavioral and Brain Sciences, Ponce Health Sciences University, Ponce 00716, Puerto Rico; ecastro@psm.edu; 5 Common Health Grounds, Rochester, NY 14607, USA; jeffrey.freeman@commongroundhealth.org; 6 Department of Surgery, University of Rochester Medical Center, Rochester, NY 14642, USA; paula_cupertino@urmc.rochester.edu; 7 Clinical and Translational Science Institute, University of Rochester Medical Center, Rochester, NY 14642, USA; 8 Department of Pediatrics, University of Rochester Medical Center, Rochester, NY 14642, USA; karen_wilson@urmc.rochester.edu

**Keywords:** e-cigarette, marketing, racial/ethnic minority, adolescents

## Abstract

Evidence suggests an association between exposure to electronic cigarette (e-cigarette) marketing and e-cigarette use (vaping) among adolescents. However, there is limited evidence on exposure to e-cigarette marketing and susceptibility to future vaping, especially among Black and Latino adolescents. This study aimed to examine associations between exposure to e-cigarette marketing and susceptibility to future vaping among Black and Latino adolescents in the United States (US). Participants (N = 362; equal representation between Black and Latino adolescents) completed a baseline assessment (available in English and Spanish) including sociodemographic characteristics (e.g., racial/ethnic group, age, gender, sexual orientation, etc.), exposure to e-cigarette marketing, and susceptibility to future vaping. Exposure to e-cigarette marketing was recoded and organized into two categories (high exposure = 2 to 3; low exposure = 0 to 1). Cochran–Mantel–Haenszel tests were used to evaluate the association between exposure to e-cigarette marketing and susceptibility to future vaping, stratified by racial/ethnic group. Multiple logistic regressions assessed the association between exposure to e-cigarette marketing and susceptibility to future vaping, controlling for gender, sexual orientation, grade, and academic performance within each racial/ethnic group. Black adolescents reported significantly higher frequencies of exposure to e-cigarette marketing (*p* = 0.005). A significant interaction was found between exposure to e-cigarette marketing and racial/ethnic group (X^2^ (1) = 6.294, *p* = 0.012). Among Black adolescents, high exposure to e-cigarette marketing (vs. low exposure) was associated with a higher probability of susceptibility to future vaping (OR: 2.399, 95% CI 1.147–5.021, *p* = 0.020). For Latino adolescents, exposure to e-cigarette marketing was not associated with susceptibility to future vaping (OR: 0.503, 95% CI 0.245–1.03, *p* = 0.062). Future studies should evaluate how and where adolescents are exposed to e-cigarette marketing. Prevention efforts must include the implementation of effective counter-marketing campaigns and the reduction of exposure to e-cigarette marketing among Black and Latino adolescents.

## 1. Introduction

In the United States (US), electronic cigarettes (e-cigarettes) have remained the most used nicotine/tobacco product among adolescents despite the decrease observed from 2022 to 2023 among high school students (14.1% to 10.0%) [1,2,3]. In 2023, 10.0% of high school and 4.6% of middle school students in the US reported using e-cigarettes [1]. Different rates have been observed according to racial and ethnic groups, with 9.7% of Latino high school students and 6.6% of Latino middle school students reporting e-cigarette use in the past 30 days [1]. In contrast, among Black adolescents, 5.6% of high school students and 5.7% of middle school students reported e-cigarette use during the past 30 days [1]. 

E-cigarette use (vaping) can impact adolescents’ physical and mental health [4,5]. There is evidence that e-cigarettes, like cigarettes, contain nicotine and various other harmful toxicants and carcinogens that have potential negative health impacts [6]. For example, individuals who exclusively use e-cigarettes have higher urine concentrations of nicotine, metals, volatile organic compounds, and tobacco-specific nitrosamines compared with nontobacco users [7]. Regarding health outcomes, in 2019, e-cigarette use, or vaping, was associated with 2807 cases of lung injury and 68 deaths in the US (e-cigarette use, or vaping, -associated lung injury: EVALI) [8]. In addition, the use of e-cigarettes by adolescents has been associated with an increased probability of respiratory symptoms, such as cough and wheezing, and health complications, including exacerbation of asthma and bronchitis symptoms [9,10,11]. E-cigarette use among youth and in animal models has been associated with an increase in pulmonary inflammation [12,13]. Furthermore, an online national survey completed in May 2020, including adolescents and young adults aged 13 to 24 years, found a significant association between COVID-19 diagnosis and the use of e-cigarettes only and their dual use with cigarettes [14]. The diagnosis of COVID-19 was higher among those who reported ever using e-cigarettes, ever using both types of smoking together (cigarettes and e-cigarettes), and ever using both for a 30-day period. COVID-19 symptoms, such as cough, fever, and difficulty breathing, were more frequent in those participants who reported 30-day dual use [14]. Vaping in adolescents has also been associated with an increased risk of becoming a combustible tobacco user, likely due to nicotine addiction [15]. 

Prior research has demonstrated an association between exposure to e-cigarette marketing and e-cigarette use [16,17,18]. A study including data from the 2014 National Youth Tobacco Survey concluded that exposure to e-cigarette marketing was significantly associated with an increased likelihood of ever and current use of e-cigarettes among middle and high school students [17]. In the same study, exposure to e-cigarette marketing was associated with an increased susceptibility to future vaping among US adolescents who did not use e-cigarettes [17]. This association between exposure to e-cigarette marketing and e-cigarette use and susceptibility to future vaping was confirmed by a recent study including national data from the 2017 to 2018 Population Assessment of Tobacco and Health Study (PATH) [18]. The study examined the association between past 30-day exposure to e-cigarette marketing in six different settings (i.e., gas stations/convenience stores, social media/websites, newspaper/magazines, radio, billboard, TV). Findings showed that past 12-month and past 30-day e-cigarette use were significantly associated with exposure to e-cigarette marketing on social media/websites (aOR = 1.65 [99.17% CI = 1.36, 1.99; aOR = 1.49 [99.17% CI = 1.13, 1.97]) and gas stations/convenience stores (aOR = 1.33; [99.17% CI = 1.11, 1.58]; aOR = 1.27 [99.17% CI = 1.03, 1.58]) [14]. Additionally, exposure to e-cigarette marketing on social media/websites (aOR = 1.35 [99.17% CI = 1.04, 1.74]) and gas stations/convenience stores (aOR = 1.67 [99.17% CI = 1.31, 2.13]) was significantly associated with lifetime e-cigarette use among baseline youth who were e-cigarette-naïve [18].

Similar results were found in a randomized trial evaluating the effect of youth-appealing e-cigarette advertising on susceptibility to e-cigarette use [19]. This study compared the effects of four e-cigarette ads with low youth appeal, four e-cigarette ads with high youth appeal, and four control ads among youth. Overall, 59% of youth in the low appeal condition and 56% in the high appeal condition were susceptible to trying an e-cigarette, compared with 46% in the control condition. Assignment to the low appeal group was associated with greater susceptibility to trying e-cigarettes compared to control, OR = 1.80, *p* = 0.03. Authors concluded that exposure to any e-cigarette advertising has an impact on adolescents’ initiation of e-cigarette use. However, the relationship with susceptibility to future vaping is limited, especially among racial and ethnic minority groups (e.g., Black and Latino adolescents). Understanding the impact of exposure to e-cigarette marketing on susceptibility to future vaping is essential to inform preventive tobacco regulatory policies. This study examines associations between exposure to e-cigarette marketing and susceptibility to future vaping among Black and Latino adolescents in the US. 

## 2. Materials and Methods

### 2.1. Study Design

This study is a secondary data analysis of a vaping prevention pilot randomized controlled trial among Black and Latino adolescents in the US. The study was approved by the Institutional Review Board of the University of Rochester Medical Center (STUDY00006267). Recruitment was conducted from August 2021 to December 2021 through proactive and reactive strategies by a team of diverse (e.g., racial/ethnic group, gender), bilingual (English and Spanish), and trained staff. The recruitment took place in three different locations in the US: (1) Hackensack, New Jersey; (2) Rochester, New York; and (3) Ponce, Puerto Rico. A convenient sample of 362 Black and Latino adolescents (N = 362; equal representation of Black and Latino adolescents) was used to estimate the effect size for future studies. The details of the clinical trial intervention, protocol, recruitment strategies, eligibility, and support details were published previously [20,21]. 

### 2.2. Measures

Participants completed a baseline assessment that included sociodemographic characteristics (e.g., racial/ethnic, age, gender, sexual orientation, language of preference, and education grade), exposure to e-cigarette marketing, susceptibility to future vaping scale, and academic performance. Baseline assessment was available in Spanish and English. 

Exposure to E-Cigarette Marketing included three questions about e-cigarette advertisements from the National Youth Tobacco Survey 2020 Questionnaire [22]. Participants were asked the following: (1) “When using the Internet, how often do you see ads or promotions for e-cigarettes?” (1 = I do not use the internet to 6 = always); (2) “How often do you see posts related to e-cigarettes when you go on social media (such as YouTube, Instagram, Snapchat, Twitter, or Facebook)?” (1 = I do not use the internet to 6 = always); and (3) “When you go to a convenience store, supermarket, or gas station, how often do you see ads or promotions for e-cigarettes?” (1 = never to 5 = always). Responses in questions one and two were recoded so that 1–4 = low exposure (0) and 5 or above = high exposure to e-cigarette marketing (1). Question three responses were recoded into 1–3 = low exposure (0) and 4 or above = high exposure to e-cigarette marketing (1). These coded responses to the three questions were then aggregated to create an overall score of exposures, coded as the sum of the number of advertising sources with positive exposure to e-cigarette marketing for each subject. This variable was organized as a binary outcome (high exposure = 2 to 3 scores of exposures; low exposure = 0 to 1 scores of exposures). 

The Susceptibility to Future Vaping Scale is a three-item questionnaire [23,24,25]. Participants answered the following questions: (1) Have you ever been curious about using e-cigarettes?, (2) Do you think you will use e-cigarettes in the next year? and (3) If one of your best friends were to offer you an e-cigarette, would you use it? The instrument uses a 4-point Likert scale ranging from not at all curious (1) to very curious (4) for question one and definitely not (1) to definitely yes (4) for questions two and three. Consistent with prior studies, categories were combined to create dichotomous variables (1 = not at all curious and definitely not; 2 = a little curious, somewhat curious, and very curious; probably not, probably yes, and definitely yes) [23,24,25,26]. Participants who provided a response other than “Definitely not” to one or more items were categorized as susceptible (Yes/No).

For Academic Performance, participants responded to the question “How would you rate your academic performance?”, with answers ranging from very good = 1 to poor = 5. To facilitate statistical analysis, the scoring was reversed from the original measure so that the larger the classification, the better the academic performance (5 = very good to 1= poor). 

### 2.3. Analysis

Cochran–Mantel–Haenszel (CMH) tests were employed to compare participant characteristics between the high and low exposure groups and to evaluate the association between exposure to e-cigarette marketing and susceptibility to future vaping stratified by racial/ethnic groups. Multiple logistic regressions were performed to assess the association between exposure to e-cigarette marketing and susceptibility to future vaping. The model was adjusted for covariates associated with susceptibility to future vaping and e-cigarette use identified in the literature (i.e., gender [27,28], sexual orientation [29], grade [30], and academic performance [31]). Interaction analysis was performed to see whether the association between exposure to e-cigarette marketing and susceptibility to future vaping was moderated by racial/ethnic group. Stratified analyses were carried out to test the association for each of the cohorts. All statistical tests were two-sided, and a p-value of less than 5% was considered significant. Statistical analyses were performed using SAS System for Windows Version 9.4 (SAS Institute Inc., Cary, NC, USA). 

## 3. Results

### 3.1. Sociodemographic Characteristics and Susceptibility to Future Vaping Differences by Exposure to E-Cigarette Marketing (High Exposure vs. Low Exposure)

Mean age of participants was 15.0 (SD = 1.5) years. More than half of the sample identified as male (n = 235, 64.9%) and heterosexual or straight (n = 317, 87.6%). Most participants listed English as their preferred language (n = 297, 82.0%). The largest groups of participants attended eleventh grade (n = 91, 25.1%) and reported very good academic performance (n = 177, 48.9%). Overall, just over half of participants (n = 198, 54.7%) were categorized as susceptible to vaping in the future. Table 1 presents a detailed description of the study participants by exposure to e-cigarette marketing group (high exposure vs. low exposure). Cochran–Mantel–Haenszel (CMH) tests results showed that Black adolescents, compared to Latino adolescents, reported significantly higher frequencies of exposure to e-cigarette marketing (high exposure = 2 to 3: 61.1% vs. 38.9%, respectively, *p* = 0.005). 

### 3.2. Associations between Exposure to E-Cigarette Marketing (High Exposure vs. Low Exposure) and Susceptibility to Future Vaping within Racial/Ethnic Group

Cochran–Mantel–Haenszel tests showed a significant association between exposure to e-cigarette marketing (high exposure 2 to 3 vs. low exposure = 0 to 1) and racial/ethnic group (X^2^ (1) = 6.294, *p* = 0.012). Thus, multiple logistic regression analysis was performed to evaluate the association between exposure to e-cigarette marketing and susceptibility to future vaping for each of the racial/ethnic groups (Table 2). Among Latino adolescents, exposure to e-cigarette marketing was not significantly associated with susceptibility to future vaping. Among Black adolescents, a statistically significant association was found: reporting high exposure (2–5) was associated with a higher probability of susceptibility to future vaping relative to less exposure (OR: 2.399, 95% CI 1.147–5.021, *p* = 0.020; Table 2) after controlling for gender, sexual orientation, grade, and academic performance. A statistically significant association between academic performance and susceptibility to future vaping was also found among Black adolescents. Better academic performance was associated with lower susceptibility to future vaping (OR. 0.272, 95% CI: 0.154–0.481, *p* < 0.0001). 

## 4. Discussion

This secondary data analysis evaluated the interaction between exposure to e-cigarette marketing and susceptibility to future vaping among a sample of Black and Latino adolescents in the US. The results reflect the potential impact of exposure to e-cigarette marketing on susceptibility to future vaping among Black adolescents. These findings echo recent research regarding the impact of exposure to e-cigarette marketing on susceptibility to future vaping among adolescents [9,27,32,33,34]. However, the results differed for the Latino adolescent group, where exposure to e-cigarette marketing was not significantly associated with susceptibility to future vaping. In addition, our findings show that, compared to Latino adolescents, Black adolescents reported significantly higher frequencies of exposure to e-cigarette marketing. These results are similar to previous studies, where differences in exposure to e-cigarette marketing were found according to race and ethnicity [18]. 

The absence of an association between exposure to e-cigarette marketing and susceptibility to future vaping among Latino adolescents could be explained by the lower exposure to e-cigarette marketing reported by Latino adolescents compared to Black adolescents. The higher exposure reported by Black adolescents could be associated with the documented perceived need to be online on social media and constantly connected to technology [35]. In a longitudinal study conducted from 2016 to 2020, Black adolescents compared to White, Latino/Hispanic, Asian, Native American, and other racial and ethnic groups reported a significantly higher need to be online on social media than other racial and ethnic groups of adolescents (e.g., Latinos) [35]. However, social media use differs from the exposure to e-cigarette marketing, which is a general approach to ads or promotions on social media, the internet, and convenience stores. 

Additionally, there is evidence that adolescents’ exposure to e-cigarette marketing at convenience stores is associated with an increase in susceptibility to e-cigarette use [18,36]. A study evaluating whether exposure to e-cigarette marketing at convenience stores was associated with susceptibility to use and future e-cigarette initiation in a national longitudinal study of youth concluded that adolescents who reported frequent convenience store access and exposure to e-cigarette marketing were at greater risk of susceptibility to future vaping [36]. Adolescents visiting convenience stores at least weekly (vs. never) had 1.51 times higher odds of e-cigarette susceptibility (95% confidence interval [CI]: 1.25, 1.81) and 1.79 times higher odds of e-cigarette initiation (95% CI: 1.29, 2.48) [36]. This is congruent with another national longitudinal study where past 12-month and past 30-day e-cigarette use was significantly associated with exposure to e-cigarette marketing on social media/websites [18]. 

Future studies should consider the aggregated impact of exposure to e-cigarette marketing in multiple environments, such as social media and convenience stores, on the risk of susceptibility to future vaping among adolescents. This is particularly important given the limited literature and differences identified in the exposure to e-cigarette marketing considering sociodemographic characteristics, such as race and ethnicity, gender, and socioeconomic status [37,38,39]. It is also important to explore other factors that may be associated with greater susceptibility to future vaping among Latino adolescents, considering that they currently have a higher frequency of e-cigarette use compared to Black adolescents [1]. For example, the role of healthcare providers may play a role in susceptibility. A recent study found that, compared to Black adolescents, Latino adolescents are significantly less likely to be advised not to use e-cigarettes by healthcare providers [39]. This could represent an opportunity to engage healthcare providers more effectively in narrowing this disparity in use. Prevention efforts should include interventions at multiple levels, not only individual, but also in the health system, such as educating and testing systems for healthcare workers to target vulnerable communities, such as racial and ethnic minorities.

Overall, the presented results contribute to the limited literature on the impact of exposure to e-cigarette marketing among adolescents by racial and ethnic groups. In addition, they highlight the need to evaluate the differences in exposure to e-cigarette marketing and its impact among adolescents from diverse racial and ethnic groups. Further research can examine additional individual and social factors associated with susceptibility to future vaping among Latino adolescents to inform future prevention and intervention actions. Such factors could include intention to use e-cigarettes, normative beliefs, social acceptability, friendship network exposure, and low socioeconomic status, which have previously been identified as significant predictors of e-cigarette use initiation and susceptibility to future vaping [38,40,41]. 

### Strengths and Limitations

This study’s strengths include evaluating susceptibility to future vaping with a measure appropriate for use among Latino and Black adolescents. Another strength is the inclusion of Spanish-speaking participants and measuring the aggregated measure of exposure to e-cigarette marketing. These strengths are crucial for developing culturally tailored interventions and prevention programs for Black and Latino adolescents in the US. 

The limitations of this study include being a secondary data analysis and its cross-sectional design, which restrict causal inference and generalizability. We acknowledge that our study, as a secondary data analysis, did not involve formal sample size estimation procedures. While efforts were made to ensure the adequacy of the sample size for examining groups separately, the absence of formal estimation methods could have implications for the generalizability and statistical power of our findings. This limitation is particularly relevant in secondary data analysis, where sample sizes are often constrained by existing datasets and may not align perfectly with the research objectives. Therefore, results should be interpreted with caution, and future studies should consider employing rigorous sample size estimation techniques to enhance the robustness of the findings. Additionally, the reliance on self-reported data for variables of interests represents a potential challenge. Exposure to e-cigarette marketing and susceptibility to self-reported data on future vaping may introduce recall bias, thereby impacting the interpretation of our results. Despite our efforts to mitigate this bias through the implementation of standardized survey instruments and clear participant instructions, there remains a need for future research to complement self-reported data with diverse methodologies, such as observational techniques, interviews, or objective metrics. This approach will enable a cross-validation of the findings, thereby enhancing the reliability and validity of the acquired data. Furthermore, the lack of a detailed description of exposure to e-cigarette marketing sources via social media (e.g., Instagram, Snapchat, or Facebook) and types of e-cigarette marketing (e.g., via ads and/or influencers) limits the interpretation of the results regarding the different impacts that could represent multiple resources and types of e-cigarette marketing. Moreover, even when there are associations identified between language and exposure to e-cigarette marketing (which is higher among English speakers), a comparison between analyses results was not included due to the small sample size of adolescents who selected Spanish as their preferred language (n = 6, Spanish vs. n = 107, English in the exposure outcome). Our results highlight the need for longitudinal studies to evaluate e-cigarette marketing’s impact on susceptibility to e-cigarette use among various racial and ethnic groups. Additionally, if language associations are stable in longitudinal studies, it is important to understand why e-cigarette marketing may not influence Latino adolescents’ susceptibility to future vaping. Future studies should evaluate additional variables that could represent protective factors for Latino adolescents from perspective of the impact of exposure to e-cigarette marketing.

## 5. Conclusions

This study provides evidence of racial and ethnic differences regarding exposure to e-cigarette marketing and its association with susceptibility to e-cigarette use among adolescents. Higher exposure to e-cigarette marketing among Black adolescents, particularly those with lower academic performance, may increase susceptibility to future vaping. However, the findings were different for Latino adolescents, where exposure to e-cigarette marketing did not demonstrate a statistically significant association with susceptibility to future vaping. The lack of a significant association between exposure to e-cigarette marketing and susceptibility to future vaping among Latino adolescents may be attributed to their comparatively lower levels of exposure to e-cigarette marketing relative to Black adolescents. Furthermore, the small sample size among Latino adolescents may have influenced the lack of significant association detected between exposure to e-cigarette marketing and susceptibility to future vaping. Restricting and reducing advertising targeted at Black adolescents may reduce the likelihood of susceptibility to e-cigarette use. Prevention efforts are necessary and must include developing and testing counter-marketing campaigns and reducing the exposure of e-cigarette marketing among Black and Latino adolescents. Moreover, efforts should be directed towards identifying and examining additional factors that could potentially be linked to the susceptibility to future vaping and explaining the observed differences among adolescents from racial and ethnic minority groups.

## Figures and Tables

**Table 1:** Sociodemographic characteristics and susceptibility to future vaping of participants by exposure to e-cigarette marketing (high exposure vs. low exposure).

Characteristic	Full Sample N = 362		High Exposure (2 to 3) N = 113		Low Exposure (0 to 1) N = 249		Significance
	n	%	n	%	n	%	*p*-Value
Racial/Ethnic Group							0.005
Black	181	50.0	69	61.1	112	45.0	
Latino	181	50.0	44	38.9	137	55.0	
Gender							0.178
Male	235	64.9	80	70.8	155	62.2	
Female	124	34.3	33	29.2	91	36.5	
Other (e.g., Transgender)	3	0.9	0	0.0	3	1.2	
Sexual Orientation							0.001
Heterosexual/Straight	317	87.6	109	96.5	208	83.5	
Homosexual/Gay	6	1.7	1	0.9	5	2.0	
Bisexual	13	3.6	1	0.9	12	4.8	
Unknown	26	7.1	2	1.8	24	9.6	
Grade							0.004
6th	4	1.1	0	0.0	4	1.6	
7th	24	6.6	2	1.8	22	8.8	
8th	43	11.9	16	14.2	27	10.8	
9th	61	16.9	14	12.4	47	18.9	
10th	82	22.7	23	20.4	59	23.7	
11th	91	25.1	35	31.0	56	22.5	
12th	57	15.7	23	20.4	34	13.7	
Academic Performance							0.087
Very good	177	48.9	68	60.2	109	43.8	
Good	130	35.9	28	24.8	102	41.0	
Average	46	12.7	14	12.4	32	12.9	
Below average	8	2.2	2	1.8	6	2.4	
Poor	1	0.3	1	0.9	0	0.0	
Language of preference							<0.0001
English	297	82.0	107	94.7	190	76.3	
Spanish	65	18.0	6	5.3	59	23.7	
Susceptibility to future vaping							0.786
No	164	45.3	50	44.2	114	45.8	
Yes	198	54.7	63	55.8	135	54.2	

Note: Exposure to e-cigarette marketing was organized into two categories (high exposure = 2 to 3; low exposure = 0 to 1).

**Table 2:** Association between exposure to e-cigarette marketing and susceptibility to future vaping within racial/ethnic group.

Variables	Susceptibility to Future Vaping (Yes, n = 198)		aOR	95% CI		*p*-Value
Hispanic/Latino	High (n, %)	Low (n,%)				
High exposure to e-cigarette marketing (vs. low) *	18, 41.0	74, 54.0	0.503	0.245	1.03	0.062
Black/African American	High (n, %)	Low (n,%)				
High exposure to e-cigarette marketing (vs. low) *	45, 65.0	61, 54.0	2.399	1.147	5.021	0.020

* Note: (1) aOR = adjusted OR. (2) Exposure to e-cigarette marketing was organized into two categories (high exposure = 2 to 3 and low exposure = 0 to 1). (3) Gender, sexual orientation, grade, and academic performance were adjusted.

## Data Availability

The datasets generated for this study are available from the corresponding author upon request.
